# Muscle-driven and torque-driven centrodes during modeled flexion of individual lumbar spines are disparate

**DOI:** 10.1007/s10237-020-01382-9

**Published:** 2020-09-16

**Authors:** Robert Rockenfeller, Andreas Müller, Nicolas Damm, Michael Kosterhon, Sven R. Kantelhardt, Rolfdieter Frank, Karin Gruber

**Affiliations:** 1 Mathematical Institute, University Koblenz-Landau, Universitätsstr. 1, 56070 Koblenz, Germany; 2 Institute for Medical Engineering and Information Processing (MTI Mittelrhein), University Koblenz-Landau, Universitätsstr. 1, 56070 Koblenz, Germany; 3 Mechanical Systems Engineering Laboratory, EMPA-Swiss Federal Laboratories for Materials Science and Technology, Ueberlandstr. 129, 8600 Dübendorf, Switzerland; 4 Department of Neurosurgery, University Medical Centre, Johannes Gutenberg-University, Langenbeckstr. 1, 55131 Mainz, Germany

**Keywords:** Axis of rotation, Finite helical axis, Confidence ellipse, Biomechanics, Spine model, Hill-type muscle model

## Abstract

Lumbar spine biomechanics during the forward-bending of the upper body (flexion) are well investigated by both in vivo and in vitro experiments. In both cases, the experimentally observed relative motion of vertebral bodies can be used to calculate the instantaneous center of rotation (ICR). The timely evolution of the ICR, the centrode, is widely utilized for validating computer models and is thought to serve as a criterion for distinguishing healthy and degenerative motion patterns. While in vivo motion can be induced by physiological active structures (muscles), in vitro spinal segments have to be driven by external torque-applying equipment such as spine testers. It is implicitly assumed that muscle-driven and torque-driven centrodes are similar. Here, however, we show that centrodes qualitatively depend on the impetus. Distinction is achieved by introducing confidence regions (ellipses) that comprise centrodes of seven individual multi-body simulation models, performing flexion with and without preload. Muscle-driven centrodes were generally directed superior–anterior and tail-shaped, while torque-driven centrodes were located in a comparably narrow region close to the center of mass of the caudal vertebrae. We thus argue that centrodes resulting from different experimental conditions ought to be compared with caution. Finally, the applicability of our method regarding the analysis of clinical syndromes and the assessment of surgical methods is discussed.

**Electronic supplementary material:**

The online version of this article (10.1007/s10237-020-01382-9) contains supplementary material, which is available to authorized users.

## Introduction

Relative movement between (lumbar) vertebrae occurs during most daily motions. In three dimensions, such a relative movement between two time instances can be represented by a *finite helical* (or *screw*) *axis* (FHA), i.e., an (instantaneous) axis of rotation that points toward the direction of possible translations. By intersecting the FHA with an anatomical plane, an *instantaneous center of rotation* (ICR) is obtained. The time evolution (path) of the ICR, the so-called *centrode*, had been investigated by multiple researchers with highly diverse and ambitious objectives, e.g. (1) recognizing general patterns in order to “give base-line references for potential diagnostic applications” (Aiyangar et al. 2017), “predicting...injurious vectors” (Qiu et al. 2003), and finding an “indicator for mechanical disorders” (Schmidt et al. 2008) or “motion characteristics of the normal lumbar spine” (Yoshioka et al. 1990), (2) evaluating “the quality, rather than the quantity, of cervical spine movement” (Baillargeon and Anderst 2013), (3) hoping for the ICR to be “interpreted in terms of...anatomical and pathological factors” (Bogduk et al. 1995), (4) describing the change in ICR location as a consequence of disk degeneration (Cossette et al. 1971; Ellingson and Nuckley 2015; Gertzbein et al. 1985), (5) attempting to relate the ICR location to the “choice of anterior and posterior instrumentation” (Haher et al. 1991) or certain implant parameters (Niosi et al. 2006), (6) demonstrating that “analysis for sagittal plane motion of the lumbar spine is possible” (Ogston et al. 1986), and (7) correlating ICR paths to facet forces (Rousseau et al. 2006). However, a recent review (Widmer et al. 2019) had revealed that, up to now, the ICR provides only faint criteria for the description of spinal kinematics under healthy and degenerative conditions.

In this work, we used elementary, individualized multi-body simulation (MBS) models of the lumbar spine (Damm et al. 2019), performing flexion movements, to introduce a statistical criterion that may serve as a first step toward describing, detecting, and eventually understanding the cause and effect of the centrode’s location: the (weighted) confidence ellipse as introduced in Sect. 2.4. The underlying assumption was that similar (individual) spinal structures, under the same loading conditions, yield a similar relative motion of segments. Of course, changes in the individual geometries cause different relative motion patterns and thus different centrodes. Yet, we show that these differences were small under the same loading condition, but distinguishable under varying conditions. Our method was utilized to identify centrode locations depending on (1) individual geometries, (2) force application modes, and (3) material properties that are influenced by clinical syndromes. First, the location of the centrode across individual spine geometries was calculated, both with and without preload representing the upper body weight. This individualized modeling is particularly worthwhile, since, on the one side, modelers from MBS and finite element (FE) communities usually employ generic models (Abouhossein et al. 2013; Qiu et al. 2003; Senteler et al. 2017; Schmidt et al. 2008), which cannot account for structure-based deviations. Experimenters, on the other side, conduct helpful individual measurements, but have no elaborated model on hand (Cossette et al. 1971; Gertzbein et al. 1985; Haher et al. 1991; Niosi et al. 2006; Ogston et al. 1986). Second, the differences in conducting physiologically-based (muscle-driven) and artificial (torque-driven) movement on the centrode were investigated, likewise with and without preload. Third, our method could aid to assess the influence of clinical syndromes or treatments on the centrode’s location, as is discussed on the example of modeling the surgical fixation of vertebrae.

## Model and methods

### Model

**Fig. 1:**
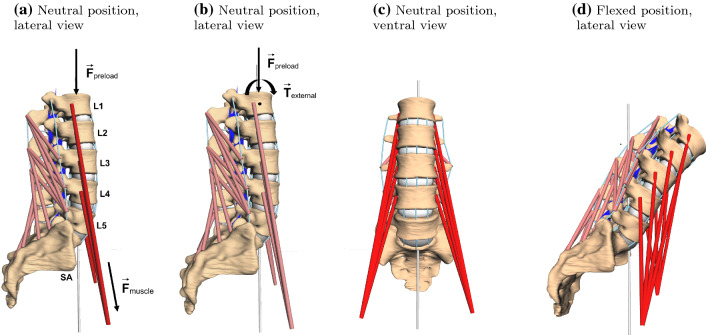
Example of an individual spine model (L1–SA) with vertebral surfaces extracted from DICOM data. Ligament (light blue lines) and muscle (red lines) insertion points were set at typical anatomic landmarks. Ligaments, intervertebral disks (gray ellipsoids) and facet joints (blue planes) transmit forces. Passive muscles are depicted in a paler red tone, whereas active muscles were depicted in vibrant red. The line of action of the preload force, representing the upper body weight, was located between the femoral heads (gravity line, silver). **a**, **b** The neutral position in lateral view, together with the acting forces ($F_{\text {preload}}$, $F_{\text {muscle}}$) and an external torque ($T_{\text {external}}$), respectively. **c** Depicts the corresponding ventral view. Slight asymmetries, resulting from individual geometries can be detected, see also Table  1. **d** A fully flexed spine resulting from applying muscle force and preload (PM), see Sects. 2.1–2.3

In total, seven individual lumbar spine models (L1–SA, cf. Fig. 1) were investigated. The term ‘individual’ here refers to the vertebral surfaces, including inter-vertebral distances, and ligament insertion points that were extracted from digital image data (DICOM). Geometries of healthy patients (six male and one female with an age of $32.6 \pm 7.04$ years) were provided by the university clinic in Mainz. After semi-automatic segmentation, vertebral surfaces were loaded into a MBS tool (SIMPACK: Dassault Systèmes, Vélizy-Villacoublay, France) under preservation of in vivo distances for intervertebral disks and facet joints. Ligament insertion points were set manually (Schünke et al. 2015) and counter-checked by neurosurgeons from the university clinic in Mainz. All passive force-transmitting structures, i.e., intervertebral disks (IVD), ligaments and facet joints, were modeled as nonlinear spring-damper elements (Damm et al. 2019). Here, particularly force-length and force-angle characteristics of ligaments and IVD, respectively, were extracted from step-wise reduction experiments (Heuer et al. 2007) and validated using data on range of motion (Heuer et al. 2007) as well as intradiscal pressure (Wilke et al. 1996). Ligaments were found to be significantly less stiff than suggested by classical data sets, e.g. (Chazal et al. 1985; Shirazi-Adl et al. 1986; White and Panjabi 1990). Indeed, recent work, combining in vivo experiments and computer simulation, support this finding (Mörl et al. 2020). Ligament pre-strain was set according to literature data, cf. (Damm et al. 2019, sect. 2.2.4).

Active forces were transmitted by Hill-type muscle models (Guenther et al. 2007; Haeufle et al. 2014; Rockenfeller and Guenther 2016, 2018) of M. psoas major and M. multifidus, each strand modeled as a one-dimensional point-to-point element with no deflection. Since neither kinematic nor kinetic data were available to conduct an elaborated parameter estimation, tendon and fiber lengths parameters were taken from literature, particularly (Christophy et al. 2012). For every muscle strand, values for optimal fiber length and tendon slack length were adapted from their Table 1, columns seven and nine, respectively. In order to maintain the fiber-to-tendon length ratio as an important functional measure (Mörl et al. 2015), both quantities were equally scaled to match individual geometries, e.g. each increased by 5% if the distance from origin to insertion was 5% higher than the literature reference. The fiber-to-tendon length ratios for each muscle strand were 2.85 for M. psoas and 2.47 for M. multifidus. For one model, exemplary optimal fiber lengths and tendon slack lengths for both muscle groups are displayed in Table 1. M. psoas maximum force was set to $80\,$N for each strand, the median value from Christophyet al. (Christophy et al. 2012, table 1, fourth column) for non-IVD parts. M. multifidus force was set to $21\,$N for each strand, likewise the median value for non-laminar parts. Remaining parameters of the muscle model were assumed to be generic and taken from (Guenther et al. 2007, table 2) (contraction dynamics) and (Rockenfeller and Guenther 2018) (activation dynamics).

**Table 1:** Optimal fiber lengths (OFL) and tendon slack lengths (TSL) of each strand within the M. psoas and M. multifidus group, exemplary for one lumbar spine model

Muscle group										
M. psoas										
Strand	Ps$\_$L1	Ps$\_$L2	Ps$\_$L3	Ps$\_$L4						
Sinister										
OFL (m)	0.212	0.184	0.156	0.127						
TSL (m)	0.0744	0.0647	0.0549	0.0448						
Dexter										
OFL (m)	0.217	0.189	0.160	0.130						
TSL (m)	0.0763	0.0336	0.0561	0.0456						
Ratio	2.85									
M. multifidus										
Strand	Mf$\_$SA$\_$L5	Mf$\_$SA$\_$L4	Mf$\_$SA$\_$L3	Mf$\_$SA$\_$L2	Mf$\_$L5$\_$L3	M$\_$L5$\_$L2	Mf$\_$L5$\_$L1	Mf$\_$L4$\_$L2	Mf$\_$L4$\_$L1	Mf$\_$L3$\_$L1
Sinister										
OFL (m)	0.0504	0.0519	0.0668	0.0864	0.0626	0.0830	0.107	0.0615	0.0837	0.0655
TSL (m)	0.0204	0.0210	0.0270	0.0350	0.0253	0.0336	0.0434	0.0249	0.0339	0.0265
Dexter										
OFL (m)	0.0523	0.0537	0.0688	0.0881	0.0644	0.0852	0.109	0.0652	0.0864	0.0661
TSL (m)	0.0212	0.0217	0.0278	0.0358	0.0261	0.0345	0.0442	0.0264	0.0350	0.0268
Ratio	2.47									

Strands from M. psoas (Ps) are labeled with the corresponding insertion point at the lumbar spine, whereas strands of the M. multifidus (Mf) are labeled with both origin and insertion, cf. Fig. 1. The listing serves the purpose of estimating the magnitudes of each muscle strand as well as demonstrating the slight asymmetry of sinister and dexter geometries. The fiber-to-tendon length ratios (OFL/TSL) for each group, which were applied for all seven models, are also given

### Calculating the instantaneous FHA and ICR during flexion

**Fig. 2:**
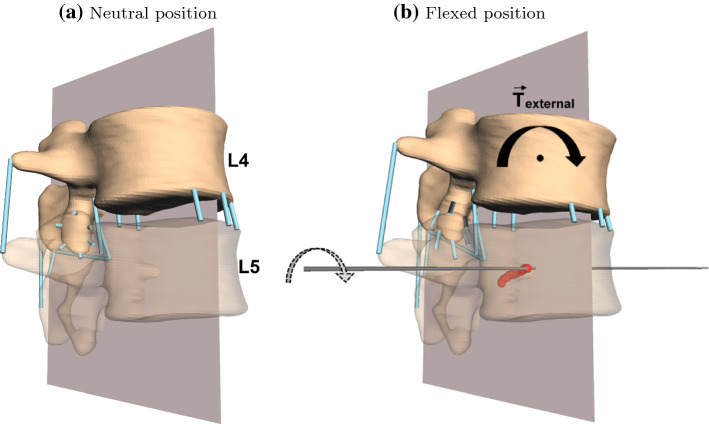
Exemplary depiction of a centrode resulting from the motion (flexion) of the vertebra L4 relative to L5. This figure is of illustrative character only. For simulated centrodes, see Figs. 3 and 4. For an animated flexion of the whole lumbar spine see the supplementary video file. **a** Neutral position of the two vertebrae L4–L5 (cf. Fig. 1a) without muscles and disk; vertebral bodies (the upper solid, the lower transparent), ligaments (blue strands) and sagittal plane (reddish gray) are depicted. **b** Flexed position of the same two vertebral bodies at the end of the movement. Besides the structures from **a**, the FHA at the final time instance (black line) as well as the intersections points of all FHA with the sagittal plane (centrode, red dots), are shown

For comparability, we hereinafter investigated only flexion of the modeled lumbar spines, i.e., the movement that occurs during forward bending of the upper body, see Fig. 1d and the supplementary video file. This flexion was driven either by muscle forces or an external torque applied at the COM of vertebra L1 (Abouhossein et al. 2013), see also next Sect. 2.3. We determined the finite helical axis (FHA) between two vertebrae using a least-squares method on spatial coordinates (Kwon 2008; Spoor and Veldpaus 1980). Therefore, four markers (ligament insertion points of ligamentum supraspinale, flavum sinister, intertransversale sinister and anterior longitudinal dexter) on each vertebral body were tracked at each time instance (every millisecond), relative to the subjacent vertebra. In a subsequent step, the intersection point of the FHA with the corresponding anatomical plane—in our case the sagittal plane—was calculated and defined to be the instantaneous center of rotation (ICR). Figure  2 depicts the situation for an exemplary L4–L5 segment. The reduction to a two-dimensional quantity is reasonable here, because the observed motion (flexion) only takes place in the sagittal plane itself. This means the vertebrae can be assumed to undergo no substantial relative translation along the helical axis, which ought to be approximately perpendicular to the sagittal plane, and thus perform a pure rotation. We further note: First, the term ‘instantaneous’ is rather to be read as ‘approximately instantaneous’ or ’finite’, because no rigorous differential-geometric approach has been applied. However, observed time intervals were small and thus this term was chosen in order to retain consistency with the literature. Second, most in vivo/vitro studies (Bogduk et al. 1995; Cossette et al. 1971; Haher et al. 1991; Yoshioka et al. 1990) and even elaborated FE models (Qiu et al. 2003; Shirazi-Adl et al. 1986; Schmidt et al. 2008) calculate only elementary, two-dimensional rotation with Reuleaux’ method (Reuleaux 1875), which is neither applicable in arbitrary three-dimensional motion nor captures translational movement of vertebrae.

### Simulation protocol: muscle-driven and torque-driven flexion

In order to investigate the differences in the paths of the ICR, when either driven by muscle (psoas) forces or 10 Nm torque, we defined four different simulation scenarios of lumbar spine flexion. Two scenarios with and two without 500 N preload, representing the upper body weight: (1) no preload and muscle-driven (NPM), (2) preload and muscle-driven (PM), (3) no preload and torque-driven (NPT), (4) preload and torque-driven (PT). Pre-load forces were supposed to act on L1 and along the gravity line (Le Huec et al. 2011), i.e., the line that vertically bisects the two femoral heads and is thought to go through the center of gravity of the upper body, cf. Fig. 1. Since CT data were obtained from decumbent patients, those scenarios were supposed to represent lying (unloaded) and standing (preloaded) positions. Torque was applied on the COM of the upmost vertebral body (L1). Total simulation time was 2.5 s in each scenario for each spine. M. psoas major started in a fully activated equilibrium ($q\approx 1$) and was fully stimulated ($\sigma =1$) for the whole simulation period, for the notation see (Rockenfeller and Guenther 2018). M. multifidus was neither activated $(q=q_0 \approx 0.01)$ nor stimulated ($\sigma =0)$ and therefore had only passive, antagonistic contributions on the ICR paths in all cases, cf. also (Zwambag,Brown 2020). Although the high stimulation value for M. psoas major, is far from physiological reality, it was chosen (a) for comparability of all simulation outputs and (b) to obtain a significant motion at all with this reduced model, see also the discussion.

### Confidence ellipses for the instantaneous center of rotation

To quantify the effects of individual geometry on ICR paths, we calculated a 95% confidence ellipse for each pair of adjacent vertebra in each of the afore-defined scenarios. The center point of the ellipses was calculated as the mean of the two-dimensional path data, and the semi-axes were represented by the two eigenvectors of the respective covariance matrix (Draper 1998; Galton 1886; Spruyt 2014). The lengths of the semi-axes thus indicate the corresponding standard deviations. We noted that variations in movement of the vertebrae were larger at the beginning than at the end of the 2.5 s simulation, because the flexed equilibrium had been reached. To account for that fact, we additionally calculated exponentially weighted confidence ellipses of the *n* (in our case $n=2500$) data points by the discrete mapping $w_{\alpha ,n}:\;\{1,\ldots ,n\} \rightarrow [0,1]$, with

1 $$\begin{aligned} w_{\alpha ,n}(k)= & {} \frac{\exp {\left( (n-k)\cdot \alpha \right) }}{\sum \nolimits _{l=1}^n\exp {\left( (n-l)\cdot \alpha \right) }} \\= & {} \frac{\exp {\left( (n-k)\cdot \alpha \right) }\cdot ( \exp (\alpha ) -1)}{\exp {(n\cdot \alpha )}-1}\;. \end{aligned}$$

Weighted mean ($\bar{\mathbf {x}}_w$) and covariance matrix ($C_w$) of the two-dimensional data $\mathbf {x}=\{(x_1,y_1)^T,\ldots ,(x_n,y_n)^T\}$ were calculated by

2 $$\begin{aligned} \bar{\mathbf {x}}_w&=\begin{pmatrix}\bar{x}\\ \bar{y}\end{pmatrix} =\sum \limits _{k=1}^n w_{\alpha ,n}(k) \cdot \begin{pmatrix}x_k\\ y_k\end{pmatrix} \end{aligned}$$

and

3 $$\begin{aligned} C_w&=\sum \limits _{k=1}^n w_{\alpha ,n}(k) \cdot \begin{pmatrix} (x_k-\bar{x})\cdot (x_k-\bar{x}) &{} (x_k-\bar{x})\cdot (y_k-\bar{y}) \\ (x_k-\bar{x})\cdot (y_k-\bar{y}) &{}(y_k-\bar{y})\cdot (y_k-\bar{y}) \end{pmatrix} \;. \end{aligned}$$

Here, $\alpha =10/n$ was found to be a suitable compromise between the location and the narrowness of the final confidence ellipses. The larger $\alpha$ the more weight is attributed to the early data points. In the non-weighted case, $w_{\alpha ,n}(k)=1/n$ for all $k\in \{1,\ldots ,n\}$.

## Results

**Fig. 3:**
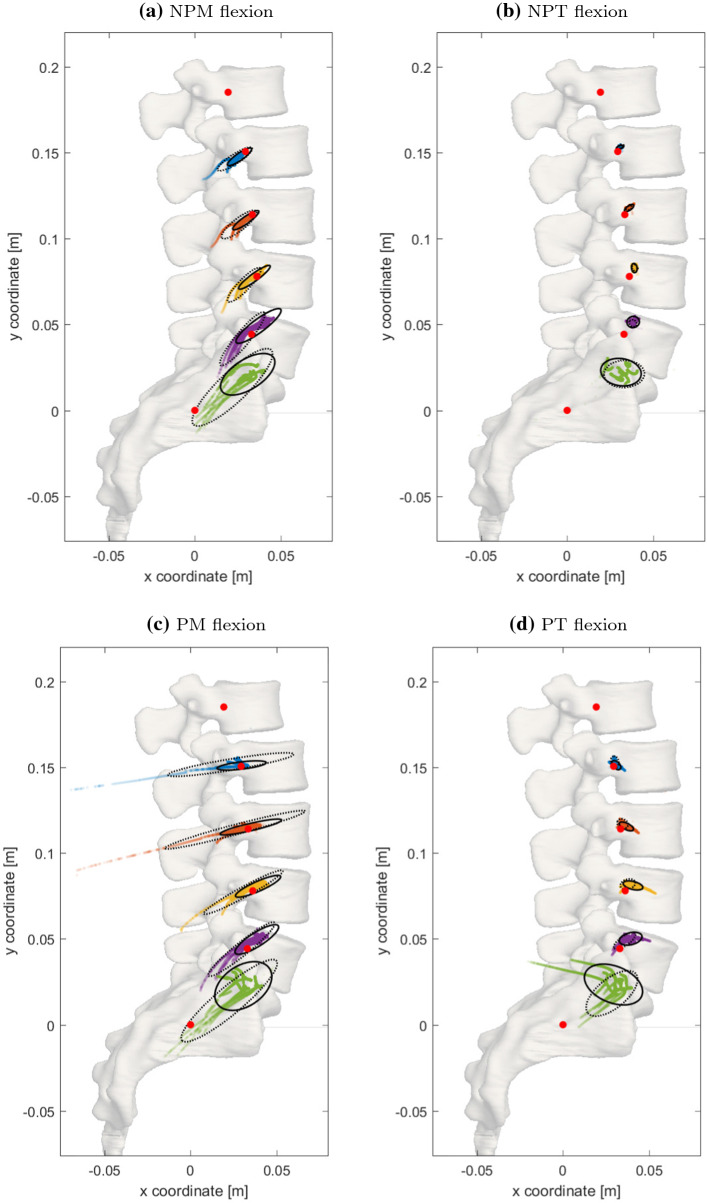
Two-dimensional locations of the instantaneous center of rotation (ICR) over time (colored dots), obtained from flexion movement of seven individual lumbar spine models. Coordinates at each instant of time were computed by intersecting the finite helical axis (FHA) with the sagittal plane. ICR coordinates were obtained relative to the center of mass (COM) of the caudal vertebra. Colors represent the spinal level (blue: L1–L2, red: L2–L3, yellow: L3–L4, violet: L4–L5, green: L5–SA). For comparability, the COMs of all seven spines at each level were superimposed (red dots). Supportive transparent vertebral surfaces are supplied in the background. These surfaces do not represent a fully physiological lumbar spine, but constitute for averaged constellations. Two types of 95% confidence ellipses for each level are shown: classical, non-weighted ellipses (solid black lines) and weighted ellipses (dotted black lines, see Eq. (1)) to capture variations in early movement. For ellipse parameters, see Table  2

Figure  3 shows the ICR-time courses between all pairs (levels) of adjacent vertebrae (L1–L2 to L5–SA) for seven individual lumbar spine MBS models during flexion. The global coordinates for the mean centers of mass were: ${\hbox {COM}}_{\text {L3}}=(25.8,119.6)$, ${\hbox {COM}}_{\text {L4}}=(32.5,83.4)$, ${\hbox {COM}}_{\text {L5}}=(31.2,48.5)$, and ${\hbox {COM}}_{\text {SA}}=(0,0)$ (units: [mm]). ICR coordinates were calculated relative to the initial positions of the centers of mass (COM) of the caudal vertebrae, which were graphically overlayed for all seven spines for easier comparison. Consequently, a ’mean’ (out of seven) lumbar spine is shown in the background of each sub-figure, not representing a real lumbar spine but constituting for visual assistance. Confidence ellipses for the non-weighted and the weighted case on each level indicate the location of 95% of all ICR coordinates across all simulated models. Table  2 provides center locations, semi-axes lengths, and orientation angle of all ellipses as well as obtained range of motion (ROM) of the corresponding lumbar spines. The following paragraphs contain a more detailed description of the four herein investigated load scenarios, i.e., PM, NPM, PT, NPT, see Sect. 2.3.

NPM Fig. 3a shows the ICR-time courses—and corresponding confidence ellipses for each level of the lumbar spines—that resulted from simulating the muscle-driven scenario without any preload (NPM). In each level of the individual, muscle-driven lumbar spine models, the ICR-time courses showed similar behavior: starting inferior-posterior to the center of mass of the caudal vertebra and moving in a superior–anterior direction. The first semi-axis lengths of the corresponding (non-weighted) ellipses increased with caudal position and were significantly longer (up to factor 5) than the second semi-axis lengths, cf. Table 2. Noticeably, the orientation angle of these ellipses did not vary substantially across spine levels ($32^\circ$–$37^\circ$). The lower the spine level (L1–L2 to L5–SA), the more ellipse centers were found to move in superior–anterior direction, with the center of the L3–L4 ellipse almost congruent to the L4 COM. Introducing the weight function (Eq. (1)) for the purpose of including early ICR data, resulted in an increase in orientation angle ($33^\circ$–$50^\circ$) as well as an increase particularly in first semi-axis lengths. ROM values from $5.1^\circ$ to $14.5^\circ$ were found comparable to the NPT scenario.

NPT Fig. 3b shows the ICR-time courses that resulted from simulating the torque-driven scenario without any preload (NPT). In any level of the torque-driven spine models, with exception of L5–SA, the ICR-time courses exhibited a similar behavior: for the whole time horizon of simulation, the ICR was located in a narrow region, superior–anterior and close to the COM of the caudal vertebra. Consequently, the semi-axis lengths were distinctly shorter than in the muscle-driven scenarios and the quotients between the first and second semi-axis lengths were substantially smaller, cf. Table 2. Eventually, this quotients became approximately 1 on the L4–L5 level, transforming the ellipses almost into circles. Contrary to the muscle-driven scenarios, in the NPT scenario the weighted ellipses were even narrower than the non-weighted ellipses, indicating that the variation in the centrode’s location took place well after the beginning of simulation. Due to the narrowness and the resulting indistinguishability of the two semi-axes, the orientation angles became a random number on the interval $-90^\circ \ldots +90^\circ$. ROM values of around $10.7^\circ$ were already mentioned to be comparable to the NPM scenario, but also matched the literature data of cadaver experiments (Heuer et al. 2007, fig. 4).

PM Fig. 3c shows the ICR-time courses that resulted from simulating the muscle-driven scenario with a preload of 500 N (PM) acting on L1 and along the gravity line, as described in Sect. 2.3. The different levels of the individual loaded lumbar spine models showed a comparable ICR-time courses as in the NPM mode, i.e., the ICR-time courses started inferior-posterior to the COM of the caudal vertebra and moved in a superior–anterior direction. Likewise, the lower the spine level, the more ellipse centers moved in superior–anterior direction, here with the center of the L2–L3 ellipse almost congruent to the L3 COM. However, in levels L1–L2 and L2–L3, few early ICR data points lay markedly posterior to the COM, thus forming a tail-shaped path toward the ellipse center. Consequently, the respective first semi-axis lengths of the weighted ellipses were longer than for the non-weighted ellipses, and for both cases longer in the PM than in the NPM scenario. Yet, the width of the ellipses (the second semi-axis lengths) showed no such differences and likewise increased in caudal direction, cf. Table  2. Ellipse centers for the PM scenario were found to be near to those from the NPM scenario. The orientation angle of the non-weighted and weighted ellipses increased in caudal direction from $7^\circ$ and $9^\circ$ to $33^\circ$ and $40^\circ$, respectively. The latter angles (L5–SA) were thus similar to the NPM scenario. The ROM ranged from $25.5^\circ$ to $39.1^\circ$ and was comparable to the ROM of the PT scenario. Furthermore, the ROM was about four times larger in the scenarios with than without preload.

PT Fig. 3d shows the ICR-time courses that resulted from simulating the torque-driven scenario with a preload of 500 N (PT). Comparable to the NPT scenario, each level of the PT models yielded similar centrodes, which were located in a narrow region, superior–anterior and close to the COM of the caudal vertebra. Here, the lengths of the first and second semi-axes were on average 1.5–2 times longer than in the NPT scenario, but still small compared to the muscle-driven scenarios. As in the NPT scenario, the range of quotients between the first and second semi-axis lengths was smaller in NPM ($1.4\ldots 2.7$) than in the muscle-driven scenarios ($1.5\ldots 14$). In addition, there was no noticeable difference between the semi-axis lengths of the weighted and non-weighted ellipses. Contrary to the muscle-driven scenarios, the orientation angles of the non-weighted ellipses were negative, except for the level L4–L5. The orientation angles of the weighted ellipses increased, as in the PM scenario, in a caudal direction from $-78^\circ$ to $39^\circ$. ROM values between $32.6^\circ$ and $41.6^\circ$ were comparable to the PM scenario. In both NPM and NPT, the size of the ellipses increases in caudal direction, indicating an amplified translation instead of pure rotation (the narrower the ellipse, the purer the rotation).

**Table 2:** ICR confidence ellipse parameters for muscle- and torque-driven flexion scenarios with and without preload (column one: NPM, NPT, PM, PT) as displayed in Fig. 3

Scenario	$$\begin{array}{l} {\text {ROM}} \\ \text {mean} \pm {\text {SD}} \\ {[{\text {min}}\ldots {\text {max}}]} \end{array}$$	Level	Non-weighted ellipse			Weighted ellipse		
			$$\begin{array}{l} {\text {Ellipse}} \\ {\text {center [mm]}} \end{array}$$	$$\begin{array}{l} {\text {Semi-axes}} \\ {\text {length [mm]}} \end{array}$$	$$\begin{array}{l} {\text {Orientation}} \\ {\text {angle}} \end{array}$$	$$\begin{array}{l} {\text {Ellipse}} \\ {\text {center [mm]}} \end{array}$$	$$\begin{array}{l} {\text {Semi-axes}} \\ {\text {length [mm]}} \end{array}$$	$$\begin{array}{l} {{\text {Orientation}}} \\ {\text {angle}} \end{array}$$
NPM	$8.2^\circ \pm 3.0^\circ$ ${[5.1^\circ \ldots 14.5^\circ ]}$	L1–L2	$$\begin{pmatrix} 26.0 \\ 147.5 \end{pmatrix}$$	$$\begin{array}{l} a=8.5 \\ b=2.1 \end{array}$$	$35^\circ$	$$\begin{pmatrix} 23.3 \\ 146.2 \end{pmatrix}$$	$$\begin{array}{l} a=11.9 \\ b=2.8 \end{array}$$	$33^\circ$
		L2–L3	$$\begin{pmatrix} 29.7 \\ 110.7 \end{pmatrix}$$	$$\begin{array}{l} a=9.3 \\ b=2.1 \end{array}$$	$35^\circ$	$$\begin{pmatrix} 25.8 \\ 108.2 \end{pmatrix}$$	$$\begin{array}{l} a=12.7 \\ b=3.6 \end{array}$$	$38^\circ$
		L3–L4	$$\begin{pmatrix} 34.7 \\ 77.5 \end{pmatrix}$$	$$\begin{array}{l} a=11.4 \\ b=2.2 \end{array}$$	$36^\circ$	$$\begin{pmatrix} 28.8 \\ 73.1 \end{pmatrix}$$	$$\begin{array}{l} a=13.6 \\ b=3.4 \end{array}$$	$46^\circ$
		L4–L5	$$\begin{pmatrix} 37.1 \\ 49.2 \end{pmatrix}$$	$$\begin{array}{l} a=16.2 \\ b=3.8 \end{array}$$	$37^\circ$	$$\begin{pmatrix} 27.8 \\ 41.7 \end{pmatrix}$$	$$\begin{array}{l} a=20.0 \\ b=4.1 \end{array}$$	$50^\circ$
		L5–SA	$$\begin{pmatrix} 30.8 \\ 21.0 \end{pmatrix}$$	$$\begin{array}{l} a=18.0 \\ b=8.7 \end{array}$$	$32^\circ$	$$\begin{pmatrix} 21.9 \\ 13.4 \end{pmatrix}$$	$$\begin{array}{l} a=31.4 \\ b=8.6 \end{array}$$	$44^\circ$
NPT	$10.7^\circ \pm 1.2^\circ$ ${[9.1^\circ \ldots 12.9^\circ ]}$	L1–L2	$$\begin{pmatrix} 30.8 \\ 153.0 \end{pmatrix}$$	$$\begin{array}{l} a=2.3 \\ b=0.9 \end{array}$$	$21^\circ$	$$\begin{pmatrix} 30.9 \\ 153.6 \end{pmatrix}$$	$$\begin{array}{l} a=2.1 \\ b=0.9 \end{array}$$	$20^\circ$
		L2–L3	$$\begin{pmatrix} 36.1 \\ 117.8 \end{pmatrix}$$	$$\begin{array}{l} a=3.1 \\ b=1.3 \end{array}$$	$33^\circ$	$$\begin{pmatrix} 35.9 \\ 117.9 \end{pmatrix}$$	$$\begin{array}{l} a=1.8 \\ b=1.1 \end{array}$$	$18^\circ$
		L3–L4	$$\begin{pmatrix} 39.1 \\ 82.9 \end{pmatrix}$$	$$\begin{array}{l} a=2.7 \\ b=1.6 \end{array}$$	$-83^\circ$	$$\begin{pmatrix} 39.4 \\ 83.0 \end{pmatrix}$$	$$\begin{array}{l} a=1.9 \\ b=1.6 \end{array}$$	$-42^\circ$
		L4–L5	$$\begin{pmatrix} 38.4 \\ 51.5 \end{pmatrix}$$	$$\begin{array}{l} a=3.6 \\ b=3.3 \end{array}$$	$13^\circ$	$$\begin{pmatrix} 38.4 \\ 50.7 \end{pmatrix}$$	$$\begin{array}{l} a=2.3 \\ b=1.9 \end{array}$$	$-35^\circ$
		L5–SA	$$\begin{pmatrix} 31.3 \\ 22.3 \end{pmatrix}$$	$$\begin{array}{l} a=11.9 \\ b=7.9 \end{array}$$	$-8^\circ$	$$\begin{pmatrix} 33.2 \\ 21.2 \end{pmatrix}$$	$$\begin{array}{l} a=11.8 \\ b=7.9 \end{array}$$	$2^\circ$
PM	$35.1^\circ \pm 4.1^\circ$ ${[25.5^\circ \ldots 39.1^\circ ]}$	L1–L2	$$\begin{pmatrix} 29.7 \\ 151.0 \end{pmatrix}$$	$$\begin{array}{l} a=14.4 \\ b=2.1 \end{array}$$	$7^\circ$	$$\begin{pmatrix} 23.4 \\ 151.6 \end{pmatrix}$$	$$\begin{array}{l} a=37.1 \\ b=3.2 \end{array}$$	$9^\circ$
		L2–L3	$$\begin{pmatrix} 35.1 \\ 115.2 \end{pmatrix}$$	$$\begin{array}{l} a=18.4 \\ b=2.2 \end{array}$$	$13^\circ$	$$\begin{pmatrix} 25.9 \\ 113.6 \end{pmatrix}$$	$$\begin{array}{l} a=42.2 \\ b=2.9 \end{array}$$	$15^\circ$
		L3–L4	$$\begin{pmatrix} 39.3 \\ 80.6 \end{pmatrix}$$	$$\begin{array}{l} a=14.3 \\ b=2.9 \end{array}$$	$24^\circ$	$$\begin{pmatrix} 30.7 \\ 77.1 \end{pmatrix}$$	$$\begin{array}{l} a=26.2 \\ b=3.0 \end{array}$$	$29^\circ$
		L4–L5	$$\begin{pmatrix} 38.6 \\ 49.6 \end{pmatrix}$$	$$\begin{array}{l} a=14.8 \\ b=4.0 \end{array}$$	$32^\circ$	$$\begin{pmatrix} 29.7 \\ 44.0 \end{pmatrix}$$	$$\begin{array}{l} a=24.7 \\ b=3.8 \end{array}$$	$39^\circ$
		L5–SA	$$\begin{pmatrix} 30.7 \\ 22.5 \end{pmatrix}$$	$$\begin{array}{l} a=18.3 \\ b=11.8 \end{array}$$	$34^\circ$	$$\begin{pmatrix} 22.5 \\ 14.0 \end{pmatrix}$$	$$\begin{array}{l} a=36.0 \\ b=7.9 \end{array}$$	$40^\circ$
PT	$37.2^\circ \pm 3.1^\circ$ ${[32.6^\circ \ldots 41.6^\circ ]}$	L1–L2	$$\begin{pmatrix} 31.0 \\ 151.2 \end{pmatrix}$$	$$\begin{array}{l} a=3.2 \\ b=1.9 \end{array}$$	$-44^\circ$	$$\begin{pmatrix} 30.1 \\ 153.1 \end{pmatrix}$$	$$\begin{array}{l} a=3.3 \\ b=2.3 \end{array}$$	$-78^\circ$
		L2–L3	$$\begin{pmatrix} 36.9 \\ 115.5 \end{pmatrix}$$	$$\begin{array}{l} a=4.5 \\ b=1.9 \end{array}$$	$-27^\circ$	$$\begin{pmatrix} 34.7 \\ 117.1 \end{pmatrix}$$	$$\begin{array}{l} a=3.3 \\ b=2.7 \end{array}$$	$-28^\circ$
		L3–L4	$$\begin{pmatrix} 40.8 \\ 81.1 \end{pmatrix}$$	$$\begin{array}{l} a=5.9 \\ b=2.3 \end{array}$$	$-14^\circ$	$$\begin{pmatrix} 37.8 \\ 81.8 \end{pmatrix}$$	$$\begin{array}{l} a=4.5 \\ b=2.9 \end{array}$$	$16^\circ$
		L4–L5	$$\begin{pmatrix} 39.4 \\ 50.1 \end{pmatrix}$$	$$\begin{array}{l} a=6.8 \\ b=3.5 \end{array}$$	$14^\circ$	$$\begin{pmatrix} 35.9 \\ 48.9 \end{pmatrix}$$	$$\begin{array}{l} a=7.4 \\ b=2.7 \end{array}$$	$35^\circ$
		L5–SA	$$\begin{pmatrix} 29.4 \\ 23.4 \end{pmatrix}$$	$$\begin{array}{l} a=17.8 \\ b=10.6 \end{array}$$	$-22^\circ$	$$\begin{pmatrix} 28.5 \\ 18.2 \end{pmatrix}$$	$$\begin{array}{l}a=18.0 \\ b=8.6 \end{array}$$	$39^\circ$

Column two: range of motion (ROM) for the whole lumbar spine models. Columns 4–9: center points, first and second semi-axes lengths, as well as orientation angles for the non-weighted (columns 4–6) and weighted (columns 7–9) ellipses at each spinal level (L1–L2 to L5–SA)

## Discussion

### On torque-driven experiments and physiological insights

Bending forward, reaching for a crate of beer and lifting it up incorrectly may result in high loading peaks within the lumbar spine (Nachemson 1965; Wilke et al. 1999). Due to unfavorable lever arms with regard to the joints, the gravitational forces of the body parts can cause high torques within the human body, which must be compensated for by the muscles. As a consequence, the internal muscle forces, transmitted according to Newton’s law (actio=reactio), eventually generate high compressive and shear forces in the various spinal structures. When experimentally isolating certain structures (e.g. vertebrae, IVDs and ligaments) while leaving others out (e.g. muscles), experimental observations may greatly differ from physiological reality. For example, isolated (lumbar) spines had been observed to “buckle” under compressive loads not even close to in vivo magnitudes (Crisco 1989). To compensate for the missing supporting structures, when applying high loads on cadaver specimen, the concept of *follower load* was established (Patwardhan et al. 1999; Rohlmann et al. 2009a). At this, a guiding rail ought to ensure a purely compressive force transmission and prevent the occurrence of shear forces, which would lead to buckling. Consequently, the forces were applied “tangent to the spinal curve, passing through the center of rotation of each segment” (Patwardhan et al. 1999, fig. 1), which was supposed to be located perfectly in between the vertebrae. It was found that the path of the follower load influences the model output and should be optimized in a sense that it passes through the centers of rotation between vertebrae (Dreischarf et al. 2010).

In both in vitro experiment and follower-load model, torque had not been physiologically induced by muscle forces, but artificially “applied” by spine testers to induce a flexion movement. We herein revealed that this method differs substantially from muscle-driven movements by means of the corresponding centrodes: torque-driven centrodes, regardless the individual spine geometry, can be found in a narrow region superior–anterior to the caudal vertebra’s COM. Muscle-driven centrodes show a more individual behavior and stretch over a wider range. These observations hold true for non-preload and preload scenarios alike, under our model assumptions. Of course, our presented model is far from capturing every physiological aspect of in vivo force transmission, since we omitted most of the whole body’s structures. However, our approach may serve as a starting point for pursuing centrode-based investigations. For example, the herein introduced concept of confidence ellipses may be utilized to assess the influence of model parameter changes (sensitivities) on the centrode location. These investigations could include the influence of ligament stiffness or failure (Abouhossein et al. 2013; Alapan et al. 2013; Putzer et al. 2016), joint forces (Senteler et al. 2017), implant positioning (Dreischarf et al. 2015; Rohlmann et al. 2010) or variable load application (Rohlmann et al. 2009b).

On the one hand, our findings support the application of the follower load concept for recreating in vitro experiments: torque-induced centrodes (and thus the path of the follower load) are virtually inert to individual geometries, instants of time, or loading scenarios. Hence, once established, the follower load can remain unchanged during the whole simulation. On the other hand, our findings speak against the application of the follower load concept for recreating in vivo experiments: the ICR location is known to change during physiological motion (Aiyangar et al. 2017, fig. 2), as is likewise visible in our Fig. 3a, c. This change in ICR location indicates the existence of translational rather than purely rotational movements of vertebrae relative to each other, which cannot be captured while utilizing a follower load.

Summarizing, our findings suggest that modeled spinal motion have to be compared with caution regarding their impetus. When aiming for physiological insights, muscle-driven models ought to be utilized. Here, it might be worth investigating whether the method of muscle control, e.g. inverse dynamic (Happee 1994), EMG-driven (Lloyd and Besier 2003) or forward dynamic (Rupp et al. 2015), has significant influence on the respective centrodes. Likewise, incorporating muscle deflection on larger muscles (Hammer et al. 2019) might lead to altered centrodes. When aiming at recreating in vitro experiments that require stabilizing follower load, torque-driven models constitute a more natural choice, but their results cannot directly be transferred to physiological reality, as statements on possible medical implications (see the Introduction and the next Sect. 4.2) might not satisfy the expectation.

### Centrodes from a medical point of view

The study of spinal motion is of utmost importance when aiming to understand the formation of disorders and the effects of surgical interventions. In clinical practice, hypermobile segments or degenerative structures commonly undergo fixing procedures. Stabilizing only one or few segments is hereby generally considered successful, although the individual spinal motion pattern is not examined in detail. If long constructs are required, or when aiming to preserve or restore “healthy” motion by application of dynamic implants (motion preserving implants), an exact balance of the resulting forces and thus profound knowledge of the motion pattern is, however, mandatory. Numerous research on motion patterns has been conducted on the cervical spine (Amevo et al. 1991; Anderst et al. 2015; Wachowski et al. 2017). This area is not only less complex than the lumbar area (due to less soft tissue involved), but also the region where most dynamic implants—especially disk prostheses—are used. The highest loads and consequently the location where degenerative changes occur first, is however be found the lumbar spine (Auerbach et al. 2008).

Changes in (lumbar) spinal kinematics have been observed following surgical procedures (spondylodesis using different techniques (Nomoto et al. 2019), facetectomy (Zeng et al. 2017), implantation of disk prothesis (Yue et al. 2019) or pedicle screw-based dynamic implants (Prud’homme et al. 2015)), but do also occur naturally due to degeneration or trauma (Amevo et al. 1992) as well as in obese patients (Rodriguez-Martinez et al. 2016). In addition, several studies, in vitro and in vivo, have been conducted to analyze lumbar spinal kinematics and to determine the centrode under healthy and degenerative conditions, see (Widmer et al. 2019) for a review. So far, however, there exists neither mechanistic nor statistical criteria linking the mere observation to a quantitative kinematic assessment, let alone to predicting the effects of surgical interventions. Given sufficient experimental data, the herein presented concept of confidence ellipses could help correlating centrodes to their corresponding clinical syndromes or medical treatments.

CT scans and bending fluoroscopy are generally available for most spinal patients. These image data allow for example to assess the grade of disk degeneration (Quint and Wilke 2008), but other structural properties—as the individual stiffness of certain ligaments or the strength of supporting muscles—can only be approximated. Further evaluation of medical image data, e.g. water-fat MRI (Schlaeger et al. 2018), could provide estimates for individualized muscle parameters, such as the maximum force. Further, stereo X-ray films (Aiyangar et al. 2017) could allow for a preciser tracing of the ICR location. Overall, the utilization of individual data constitutes an important step toward an individual spine model that could eventually be used to predict the effect of surgical interventions and to optimize operative plans before surgery (Kantelhardt et al. 2015). Implants and their positions could thus be selected, not solely but among others, on basis of individual centrode simulations, see also the next section.

### Consecutive fixation systematically alters the centrode: an exemplary scenario

Modeling load changes in lumbar spines, as a result of surgical implant placement, are utilized to assess the required medical procedure a priori (Xu et al. 2019). Additional criteria could be derived by classifying the respective centrode paths. In the literature, however, we identified two major issues in existing studies. First, lumbar spine models (Kiapour et al. 2012), which are validated using in vivo data (Pearcy and Bogduk 1988), where the center of rotation had been calculated only between the start and the endpoint of the movement, omit all mid-range dynamical information (Dombrowski et al. 2018). Second, models (Abouhossein et al. 2013), which are validated using torque-driven in vitro data (Rousseau et al. 2006), where one of the vertebrae had been fixed, obtain centrodes in between the vertebrae, which is contrary to in vivo findings (Aiyangar et al. 2017, fig. 2). These two issues can also be found combined (Naserkhaki et al. 2018; Schmidt et al. 2008).

**Fig. 4:**
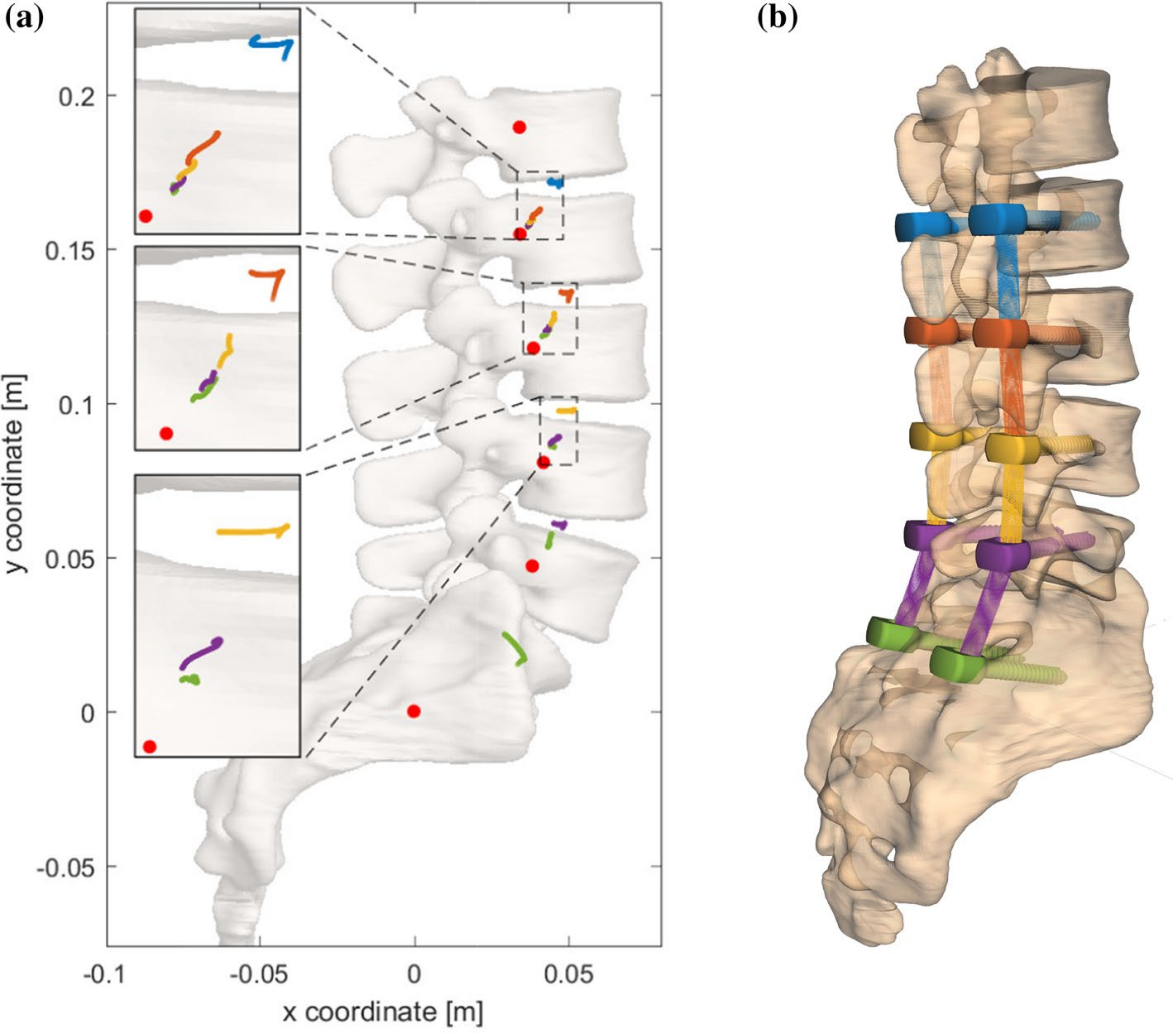
**a** Two-dimensional centrode locations for five consecutive degrees of fixation (colored dots), obtained from running NPT scenarios on an individual lumbar spine model. The lateral view on the lumbar spine is shown in the background and crucial regions are highlighted by a zoom. **b** Semi-transparent three-dimensional depiction of the corresponding implant (or pedicle screw) placements within the vertebral bodies, without further active or passive structures visible. Same colors indicate the same degree of fixation: (1) SA–SA (green, standard NPT as in Fig. 3b), (2) SA–L5 (violet), (3) SA–L4 (yellow), (4) SA–L3 (red), (5) SA–L2 (blue). ICR coordinates were computed as described in Sect. 2.2

In this section, we cursory glance at possible consequences of consecutively fixating spinal segments, i.e., inserting rigid implants starting from the sacrum and moving cranially up to L2. Figure  4 visualize the scenario and the results from a single NPT model that was utilized for a purely exemplary purpose, thus no error ellipses were calculated. Although we stated above that muscle-driven models ought to be consulted when aiming at confidable physiological findings, more sophisticated structures are required in our model to allow for significant ROM in the ultimately fixated scenario. Let SA-*X* denote a fixation from the SA upward to segment *X*, i.e., SA–L2 refers to a spinal unit where SA to L2 are rigidly connected by implants. The ROM of the flexion induced by 10 Nm expectedly decreased during consecutive fixation: from ${\hbox {ROM}}_{\text {SA--SA}}=10.5^\circ$ to ${\hbox {ROM}}_{\text {SA--L2}}=0.3^\circ$. The corresponding centrodes showed a systematic behavior: the further away segments were from the fixation, the narrower and more inferior-posterior the centrodes were located, ultimately approaching the COM of the caudal body. Within these centrodes, the migration path of the ICR was found to evolve superior–anterior. The segments, which were located directly cranial to the fixation showed a hook-shaped centrode right in the middle of the respective IVD.

These findings, although conducted with a rather simplified model, suggest a critical view on the aforementioned issues: mid-range dynamics as well as multi-($>2$)-body analyses could play a crucial role in assessing operational methods such as optimizing implant positioning (Haher et al. 1991; Niosi et al. 2006).

## Supplementary material

Three video files showing an exemplary flexion movement, from neutral position to full bending (cf. Fig. 1a, d), are provided. Each video depicts a different view on the lumbar spine (frontal, lateral and lateral-frontal). At every time instance, the corresponding FHA are shown.

## Electronic supplementary material

Below is the link to the electronic supplementary material.

Supplementary material 1 (mpg 35493 KB)

Supplementary material 2 (mpg 37245 KB)

Supplementary material 3 (mpg 32321 KB)
